# How to Find Vacant Green Space in the Process of Urban Park Planning: Case Study in Ningbo (China)

**DOI:** 10.3390/ijerph17218282

**Published:** 2020-11-09

**Authors:** Shunwei Ji, Renfeng Ma, Liyan Ren, Caijuan Wang

**Affiliations:** 1 Department of Geography and Spatial Information Techniques, Faculty of Geography, Tourism and Culture, Ningbo University, Ningbo 315211, China; 1911073006@nbu.edu.cn (S.J.); wcj18834406903@163.com (C.W.); 2 Ningbo Universities Collaborative Innovation Center for Land and Marine Spatial Utilization and Governance Research, Ningbo University, Ningbo 315211, China; 3 Institute of East China Sea, Ningbo University, Ningbo 315211, China

**Keywords:** park green space, residential area, accessibility, green space allocation, spatiotemporal matching, Ningbo

## Abstract

Nature-based recreation in urban areas is essential for the well-being of citizens. Park green space (PGS) is a necessary urban infrastructure and a critical step of urban planning and policy-making. The existing research on PGS only focuses on service allocation problems existing in the current urban development, ignoring changes in residential communities accessibility. This research provides new ideas to evaluate PGS. Based on parks and residential communities’ data, we adopt an improved Gaussian-based two-step floating catchment area (2SFCA) method to evaluate PGS accessibility in Ningbo (China) and its matching with different levels of residential areas. We present a case study in Ningbo, and discuss its implications for PGS management. This study contains two elements: (a) Compare the current and initial PGS accessibility of each community to accurately identify the communities with PGS vacancies. (b) Analyze and discuss the association between community accessibility and residential house prices. Compare the PGS coverage ratios of communities at different levels to determine the equity of PGS planning in Ningbo. We found that the level of PGS allocation in the central area of Ningbo is high. Obviously, high-value clusters are formed in Sanjiangkou, Zhenhai New Town, Southern and Eastern Yinzhou. The accessibility level in the middle area of Yinzhou is low, and there are super high accessibility residential communities in the outer city area. There is an exact period of green space vacancy in the middle and the outer area. The residential areas with ultra-high accessibility did not configure PGS services at the beginning of their construction. There is no noticeable difference in PGS accessibility of residential communities of different levels at present, but 149 low- and middle-income residential communities lack green space service when the construction was completed. High-end residential communities have priority on enjoying park green space services. Our study suggests that PGS accessibility should be studied temporally and spatially for each residential community. The Ningbo government should strengthen the balanced construction of green space in parks and guarantee green space services for low-end residential communities to improve green space equity.

## 1. Introduction

Half of the world’s population lives in cities, and the urban population’s rapid growth places higher demands on infrastructure [1]. The distribution and quality of urban green space highly affect urban residents’ physical and mental health [2,3,4]. However, due to the rapid urban expansion and population growth, planners face a significant challenge in preserving urban green spaces, especially parks in developing countries [5]. Although there are precise legal requirements for green space and public green space per capita, government measures have resulted in a mismatch between green space supply and demand, resulting in social inequity and low service satisfaction in the city [6,7]. Therefore, it is necessary to clarify the spatial differences in green space accessibility in urban planning [8,9]. This research will focus on publicly accessible park green space (PGS), which will benefit urban residents’ health and recreation.

Since the 1980s, green space research has increased, mainly using green space rate and green area to reflect the overall green service level. However, there is not enough consideration at the micro-level. In 1998, Chinese scholars first proposed landscape accessibility, which became the main content of green space research [10]. Researchers have paid more attention to the study of green space accessibility and its influencing factors [11]. For example, Cetin investigated the accessibility of green space in Kutahya and its influencing factors [12]. Lin and Yuqi found that the road network’s perfection and passage are important factors affecting PGS accessibility [13]. However, similar researches have focused on the demand for green space in the whole city, and less attention has been paid to whether the resources in each urban area match the needs of the residents. As a result, the disparity in the regional distribution of public resources at the spatial level is covered by the total PGS [14]. In reality, inequality due to socio-economic differences already exists and is exacerbated by the spatial mismatch between the population′s demand and resources [9,15].

Research on PGS equity is usually based on spatial accessibility. Euclidean distance or road network analysis in geographic information systems (GIS) is often used to examine the shortest distance to the nearest green space. However, people may choose other green spaces over the nearest one because of social preferences and PGS size. Accordingly, the gravity model is employed to address this issue. Radke and Mu first proposed a two-step floating catchment area (2SFCA) approach to measure spatial accessibility that takes into account interactions [16]. Luo further developed this method into the Enhanced 2SFCA (E2SFCA) method [17], which has been extensively studied for access to medical [18] and sports facilities [19]. This method reveals more shortage of space areas but proposes a limited solution on uniform accessibility in each catchment [20]. Dai addresses this problem by introducing a Gaussian function in 2SFCA, which effectively finds missing areas and estimates green space accessibility [21]. Therefore, this study uses the Gaussian-based 2SFCA model to measure spatial accessibility and disparity changes in Ningbo. We creatively set a threshold value to distinguish differences in accessibility. Section 2 describes the details of the methods.

Research on the equity of green spaces has focused on spatial equity and social equity. Spatial equity focuses on differences in the spatial distribution and quality of green spaces. The research found significant differences in the number of users for parks of different sizes [22]. Rigolon found that inequalities in park area and quality may exacerbate social inequalities [23]. Therefore, accessibility studies need to emphasize green space attributes’ delineation with different service ranges [24]. Social equity focuses on differences in the social attributes of urban residents [25,26,27]. Western scholars first considered the influence of racial factors in enjoying public green space services [28]. For example, it found that low-income people of color have lower green service levels than white and wealthy people [29]. Economically, the difference between the rich and the poor is also a key factor in research [30,31]. Because of the economic and ethical factors in green gentrification, the green space allocation mechanism is required to be more fair and efficient [32,33]. Chinese scholars have emphasized that there is a difference between social equity and social justice [15]. Equity means that all residents have equal access to public services. Justice allows the special provision of public services based on the differences in residents′ abilities and needs. However, because residents’ capacity and needs are difficult to quantify accurately, the accessibility is usually evaluated by the number or density of the population, so researchers pay more attention to the equal rights of residents to enjoy green space services [34,35,36]. The results of the study are not as consistent as expected. Moreover the situation varies from city to city. The location of Wuhan Park Greenland neglects the convenience of low-income people in travel [37]. Still, low-income people in Guangzhou are not as disadvantaged as expected in terms of park accessibility [38]. Whether underprivileged groups are genuinely in a disadvantaged position in the green service needs to be summarized based on the specific conditions of different cities.

In these previous studies, we found a hidden problem. The accessibility evaluation in the green space equity study focused on PGS’s service level at the present stage but neglected the PGS matching with the residential community in its development [39]. The construction of PGS takes time, and the accessibility of it must change rapidly. Traditional Chinese green space research has little content on accessibility changes, and researchers have generally focused on the allocation of green space in the current urban development process. Lijun and Yangfang examined the evolution of green space accessibility in Wuhan parks from 2000 to 2014 by delineating three temporal stages [40]. However, such a study ignores a significant issue: the changing accessibility of individual community. The accessibility of residential communities is not always in a static state, and if a park is built later than a residential neighborhood, there will inevitably be a period of vacant green spaces, which may last for years or even decades, which is unacceptable for urban dwellers. However, previous studies have not accurately calculated PGS accessibility from year to year. They tend to calculate PGS accessibility at intervals, such as once every five years and once every three years. This situation can lead to the inability to correctly identify residential communities’ accessibility. Therefore, we creatively scale green space accessibility precisely to each residential community. We define green space vacancy as when a community did not have PGS supply when it first built. By comparing the PGS accessibility of residential communities at the current stage and at the time of completion, we can accurately identify communities with periods of PGS vacancy. To further explore the issue of its fairness, we compare the community′s PGS accessibility to its housing prices and propose measures to reduce mismatch, thereby improving the quality of life for residents and informing other similar studies of urban green space planning.

Being an economic, political, and cultural center in the Yangtze River Delta China and currently experiencing unprecedented growth and expansion, Ningbo is selected for analysis in this study. Moreover, this city has experienced a tremendous green space loss, similar to many other cities in China. Therefore, we select Ningbo as the research area to enrich the local green space service research cases in China. On the research scale, Chinese studies are mostly evaluated on the sub-districts scale [41]. The evaluation of Sub-districts green space may lead to inaccurate results. It is impossible to refine green space distribution within the street, which has limited urban planning guidance. The residential community-scale is the daily recreation area of residents, and the coverage of green space in the region affects residents’ physical and mental health [42]. To increase the data’s accuracy and avoid large errors we choose residential communities as the evaluation unit and estimate the total population in the study area by combining the Ningbo 2019 statistical yearbook data. We set the service radius of different types of parks and use the improved Gaussian-based 2SFCA method to measure PGS accessibility in central Ningbo. The study focused on the following scientific questions: (1) To identify the PGS service vacancies in the city by considering the temporal and spatial matching between PGS and residential communities. (2) To explore the effects of economic level on PGS service. Our purpose is to explore whether there is a lack of PGS service in each community in the process of urban green space planning. If such a PGS service vacancy exists, whether it is caused by social injustice. We expect to provide a scientific method for PGS research and urban planning. The paper proceeds as following. The following section introduces the study area and data source. Section 3 explains the analytic and modeling framework and methods. Section 4 presents the results. Section 5 and Section 6 present the discussion and conclusions, respectively.

## 2. Study Area and Data Sources

### 2.1. Case Study: Ningbo—A Typical City in the Yangtze River Delta

**Figure 1:**
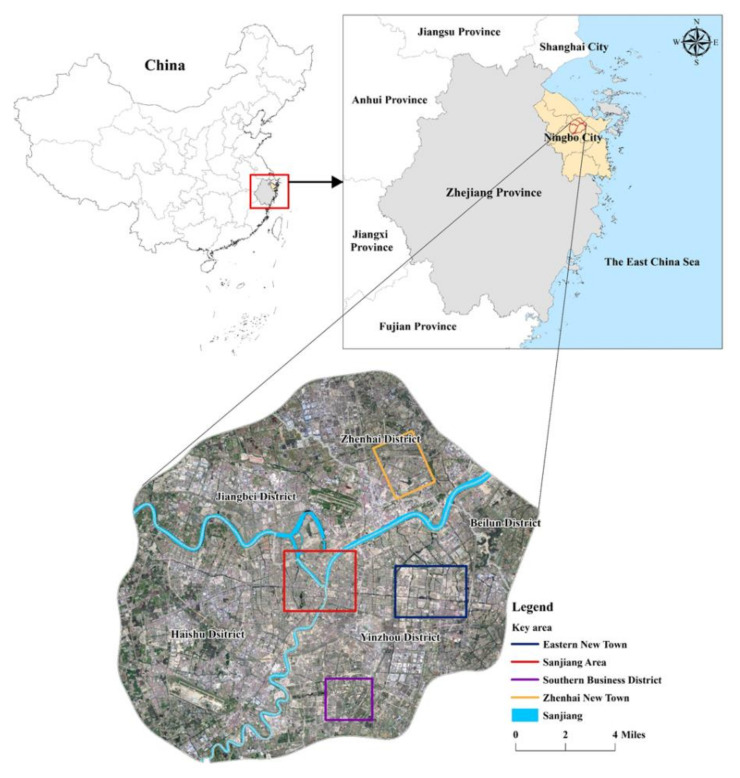
Overview of the study area.

China’s research is mostly concentrated in first-tier cities, such as Beijing [43], Shanghai [44], Hangzhou [45], Guangzhou [46], and Hong Kong [47]. After a long period of development, first-tier cities have formed a relatively complete green space system. Second-tier cities also have a longer development period, but due to the influence of policies and markets, the city’s overall public facilities lag behind first-tier cities, and the system has apparent defects. As one of China′s most livable cities, Ningbo′s PGS facilities’ spatial structure is more worthy of in-depth consideration and research. Therefore, we select Ningbo as the research area to break through the discursive power of Beijing and Shanghai and enrich the local green space service research cases [48]. Ningbo is located at the coast of the East China Sea and in the north of Zhejiang. It is the economic center of Zhejiang Province and the core city in the southern Yangtze River Delta (located at 120°55′ to 122°16′ east longitude and 28°51′ to 30°33′ north latitude). It covers 9816 square kilometers, with six districts, two counties, and two county-level cities [49]. The main urban area is composed of Haishu District, Yinzhou District, Jiangbei District, Zhenhai District, Beilun District, and Fenghua District, which are located in the middle of Ningbo City (Figure 1). Ningbo has undergone three major administrative division adjustments since 2000. In 2002, Yin County was abolished, and Yinzhou District was established. Jiangdong District was abolished and merged into Haishu District and Yinzhou District, and Fenghua changed from county to district in 2016. According to the Ningbo central urban green space system’s planning, the study area was selected, namely the central metropolitan area, where the Yongjiang, Yaojiang, and Fenghua Rivers intersect with each other (hereinafter referred to as “Sanjiang”). It is the Ningbo economic and commercial center since ancient times with the complete construction of public facilities. Simultaneously, with the Ningbo Urban Expressway’s completion, the embryonic form of the inner, middle, and outer area has taken shape. The inner area covers Haishu, Jiangbei, and Jiangdong (withdrawn area). It is surrounded by an airport road viaduct, Tongtu Road, Century Avenue, and south ring viaduct. The basic public service facilities in the inner area are relatively complete. The middle area is closely related to the transfer of the center of urban development. Yinzhou central area and the new eastern city are all located in the middle area, surrounded by an airport road viaduct, the north ring viaduct, east ring viaduct and, Yinzhou Avenue. The ring expressway covers the outer area road, the urbanization level is low, and the related infrastructure construction has been accelerated recently. It is a typical urban-rural integration area in Ningbo.

### 2.2. Data Sources and Processing

#### 2.2.1. Data of Ningbo PGS

**Figure 2:**
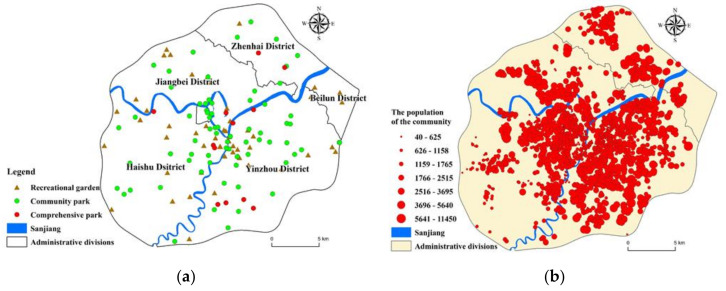
Park type (**a**) and residential community population distribution (**b**) in Ningbo.

Because of its openness and publicity, PGS has become the primary way for Chinese residents to obtain green space services. Take PGS as the service supply source, and use the park level and green space area to reflect the supply capacity. According to the “2014 Ningbo Ecological Green Space System Special Plan”, map the distribution of parks in the study area (Figure 2a), Including 12 comprehensive parks, 69 community parks, and 53 recreational gardens and using ArcGIS 10.2 to measure its area. According to China′s “urban green space classification standard,” the park is divided into different levels (Table 1), and different service radius is divided based on the park green area. The comprehensive park’s service radius is from 2000 m to 5000 m, and the service radius of the community park is 800 m and 1200 m. The service radius of the recreational garden is 300 m and 500 m. Figure 2a shows the distribution of different levels of parks in the central urban area of Ningbo. We find that the parks at the junction of Sanjiang are clustered.

#### 2.2.2. POI Data of Residential Communities

**Figure 3:**
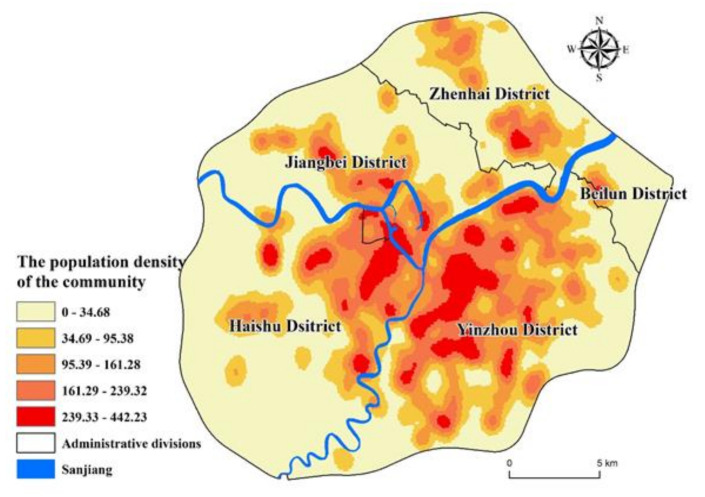
The population density of the residential community in the center of Ningbo.

Using web crawlers to collect residential community information on the websites of “Anjuke” (https://nb.anjuke.com/), “Lianjiawang” (https://nb.lianjia.com/), and “Ningbo Housing Management Bureau,” 2225 residential communities in the study area were collected. The main information includes location, number of households, average price, and year of construction. According to the “Ningbo statistical yearbook 2019”, the average population per household is 2.50. Based on this, calculate the total population of each residential community. Furthermore match the location of it on the map (Figure 2b). Using the people as the weight field to draw the population distribution density map of the study area (Figure 3), it can be seen that the people in the Sanjiang area is relatively concentrated. Ningbo’s current development center, the middle part of Yinzhou, the new eastern city, and the southern business district have become the most densely populated areas. In the northeast direction, Zhenhai’s new town also has a relatively dense population.

## 3. Methods

### 3.1. A General Overview of the Study

**Figure 4:**
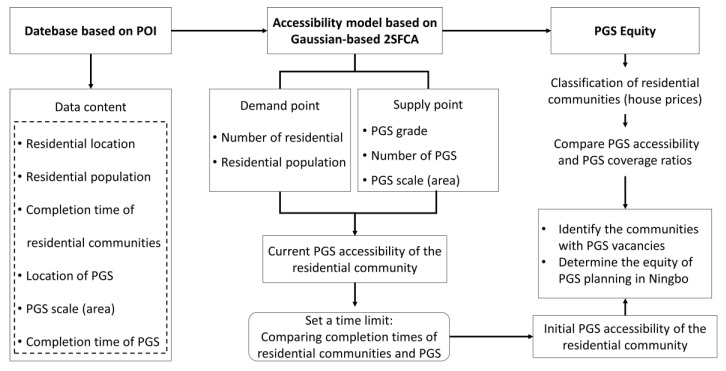
Technology flowchart.

The framework proposed in this study is schematized in Figure 4. Accessibility levels are quantified by a Gaussian-based 2SFCA model based on supply and demand. Please refer to Section 2.2 for a detailed description of the model. By setting the service radius of different PGSs, we accurately derive the supply level of PGSs in Ningbo. Combining the location and population of the residential communities, we calculate the level of PGS accessibility at the current stage. Based on this, we modified the method by setting a time limit to calculate PGS’s accessibility at the beginning of each residential community’s construction. To further explore PGS planning equity in Ningbo, we use housing prices to reflect residents’ socio-economic attributes. The study aims to provide a new perspective to evaluate the distribution of urban PGS that is easy to imitate and practice. We hope to provide new insights for evaluating spatial equity in urban public facilities and guidance for urban planning departments.

### 3.2. An Improved Gaussian-Based 2SFCA Method

The accessibility calculation method is continuously improved, from the initial shortest distance and minimum time method, to the later gravity model, two-step floating catchment area (2SFCA) method. In recent years, 2SFCA has gradually become the mainstream because of its rich expansion forms for different cities. Radke and Mu first proposed 2SFCA, and then the extension of the distance attenuation function was introduced [16]. The primary forms include enhanced 2SFCA, gravity 2SFCA, nuclear density 2SFCA, and Gaussian-based 2SFCA. The weight of each green space in the traditional 2SFCA search radius is the same, so there is an inevitable error between the ideal situation and the actual situation. The enhanced 2SFCA divides the distances into different stages within the search radius for calculation. The gravity 2SFCA adds a power function to the search radius for straight distance attenuation, and the kernel density 2SFCA adds the kernel density function for distance attenuation. The distance attenuation of Gaussian-based 2SFCA is different from the former. The attenuation of accessibility is first accelerated and then slowed down as the distance increases within the radius. Gaussian-based 2SFCA is widely used in the research of medical facilities and urban green space [20,36,50]. Ningbo urban expansion presents evident stages, and the expansion direction mostly extends eastward, mainly in the way of multi-center diffusion, and Zhenhai, Yinzhou, and Beilun are scattered and expanded. Generally speaking, the Gaussian-based 2SFCA attenuation is the most suitable for PGS research in Ningbo.

The extended form of the 2SFCA method has not been adjusted according to the research content in the current study. With the aim in mind, in this paper, we present an improved Gaussian-based 2SFCA for PGS. Add a time limit to the 2SFCA: when the park is built later than the community, it will not be counted in the service radius. Through this restriction, PGS accessibility in the present and at the beginning of the construction is calculated. By comparing the PGS accessibility at different time points, identify people with green space service vacancies. The specific process is as follows:

First of all, we set a space distance threshold *d*_0_ (the selection of the spatial distance threshold is determined according to the park’s level and its service radius) for each park green space *j* to form a spatial scope. For the community population *k* distributed in the spatial scope, we use Gaussian equation to give weight. The weighted population is summed to get all the potential users of the green space *j*, and then the green space is divided by the potential users to get the supply-demand ratio *R_j_*.

(1) $$R_{j}=\frac{S_{j}}{\sum_{k\in \left\{d_{kj}\leq d_{0}\right\}}G\left(d_{kj},\;d_{0}\right)C_{k}}$$

*C_k_* is the number of community population in the spatial scope of green space *j*; *d_kj_* is the spatial distance from the center of the community to the center of green space *j*; *S_j_* is the capacity of green space, where the area of park green space reflects the supply capacity of the park. *G*(*d_kj_,d*_0_) is the Gaussian equation, see Equation (2):

(2) $$G\left(d_{kj},d_{0}\right)=\left\{\begin{array}{c} \frac{e^{-\left(\frac{1}{2}\right)\times \left(\frac{d_{kj}}{d_{0}}\right)}-e^{-\left(\frac{1}{2}\right)}}{1-e^{-\left(\frac{1}{2}\right)}},\;if\;d_{kj}\leq d_{0}\\ 0,\;if\;d_{kj}>d_{0} \end{array}\;\;\right.$$

In the second step, for each residential community *i*, given a spatial distance threshold *d*_0_ (according to the pedestrian walking speed of 5 km/h, 30 min is the maximum walking time, so here is appropriate to take 2500 m for *d*_0_) to form another spatial scope, The supply-demand ratio of each green area *l*, *R_l_* is weighted by the Gaussian equation, and then the weighted weekly supply-demand ratio is summed to obtain the park green space accessibility of each residential community *A_i_*, the size of *A_i_* can be understood as the threshold per capita green space in each district in the area:

(3) $$A_{i}=\left\{\begin{array}{c} \sum_{l=\in \left\{d_{il}\leq d_{0}\right\}}G\left(d_{il},d_{0}\right)R_{j},\;T_{l}\leq T_{i}\\ \;0,\;T_{l}>T_{i} \end{array}\right.$$

In the formula: *R_l_* represents the supply-demand ratio of green space *l* within the spatial function threshold of residential community *i*, *T_l_* represents the opening year of green space *l*, and *T_i_* represents the construction year of residential community *i*. The description of other indicators is the same as above. The accessibility value can be understood as the per capita green area after a unique weighting process. With this formula, we can find out some communities. These communities were not served by parks and green spaces at the time they were first built. We call them unserved communities.

**Figure 5:**
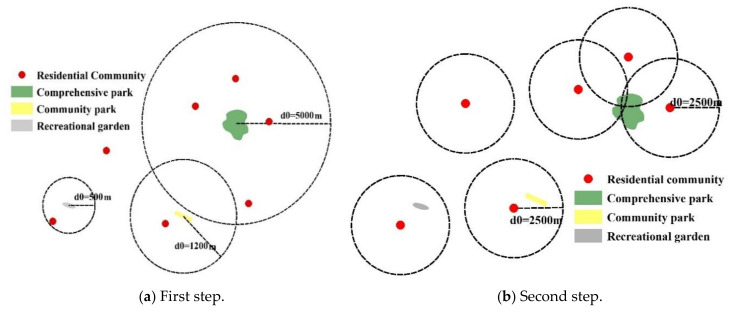
The Gaussian-based two-step floating catchment area (2SFCA) method.

Figure 5a shows an example of the first step, where we set *d*_0_ according to the PGS type, and there are six residential communities in the spatial scope of the PGS. For each of them, the spatial distance from the PGS can be measured, and then the corresponding Gaussian equation can be established according to Equation (2), respectively. The PGS area and the number of people represented by each community are also known, so the corresponding supply and demand ratios can be derived from Equation (1). In Figure 5b, we set the spatial extent with the residential community as the center, and *d*_0_ is set to 2500 m depending on the residents’ travel speed. According to Equation (3), the PGS accessibility of the residential community is obtained by accumulating the supply ratio to demand for each community.

### 3.3. Park Greenspace Equity Evaluation

The accessibility calculation result is a spatial latitude value. To make the results easy to understand and analyze, we use house prices to represent the socio-economic attributes of residents. We obtain the prices of houses in the residential neighborhoods within the study area and then match them with the accessibility. According to the average housing price, the residential communities are divided into six levels: first, second, third, fourth, fifth, and unit communities. Respond to the disparity in residents′ access to green space services by counting the number of unserved communities in different classes of neighborhoods. The ratio of green space coverage in Ningbo is based on the greening requirements of the city. Compare the current differences in urban green space coverage.

(4) $$C_{r}=\frac{N_{i}}{S_{i}}\;$$

In Equation (4), *C_r_* is the coverage ratio of park green space. *N_i_* is the number of *i*-level communities within 500 m of the park green space. *S_i_*is the total number of i-level residential communities.

## 4. Results

### 4.1. Current PGS Accessibility

**Figure 6:**
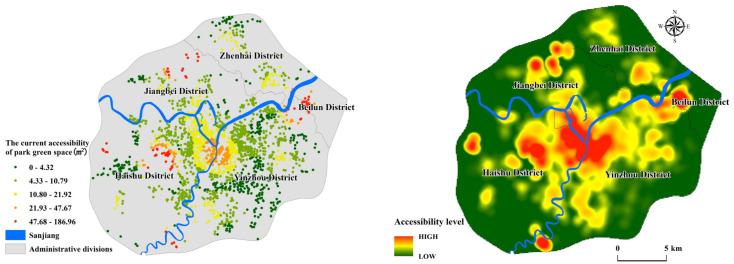
The current accessibility of PGS in Ningbo downtown area.

At present, the accessibility of PGs in Ningbo is high, but there is imbalance among regions. The Sanjiang area has high-value clusters. Zhenhai New Town, Yinzhou Southern Business District, and Eastern New Town maintain a high level. Meanwhile, there are ultra-high accessibility residential areas in the outer area (Figure 6). Sanjiang area is the area with the most extended development history in Ningbo. Compared with other regions, it has relatively perfect necessary public service facilities. Although the residential community’s population density is high, it has a large area of comprehensive parks, such as Yuehu Park, Rihu Park, and Zhongshan Park. It is the area with the best green space service level in Ningbo. Yinzhou District, as the current center of Ningbo, has the highest population density. The Southern Business District and the Eastern New Town maintain good accessibility. The Southern Business District has three comprehensive parks, Tiangong Manor, Yinzhou Park, and Academician Park. There are many community parks in Eastern New Town, such as science and technology parks and citizen squares. However, Figure 6 shows that the service level of green space in the middle part of Yinzhou is low. This area maintains a high population density. However, due to the lack of large-scale comprehensive parks, the accessibility here is low. Only Binhe Park, Yujin Park, and Qianyindong Park are distributed; these community parks cannot meet residents’ green space needs.

There are few parks in Zhenhai District. However, two high-value gathering areas are evident. High-access residential communities are mainly distributed near the Ningbo Botanical Garden and Zhenhai Green Axis Sports Park. Because this is the central area in the Zhenhai region. Ningbo Botanical Garden exceeds 10 hectares, which is significantly improving the service level of PGS in surrounding areas. Surprisingly, some residential communities in the outer area of the city have extremely high PGS accessibility. Combined with the population density map, we find that the super high accessibility residential areas in the city’s outer area are its low population density. There is pretty good PGS scattered around them. Taking Beilun ultra-high accessibility level residential areas as an example, the region’s total population is only. There are seven community parks within its radius of 2.5 km, including Xinmo Park, Meixu Park, Ningbobang Park, and Dadongjiang park. The total green area is more than 20 hectares.

### 4.2. Initial PGS Accessibility

**Figure 7:**
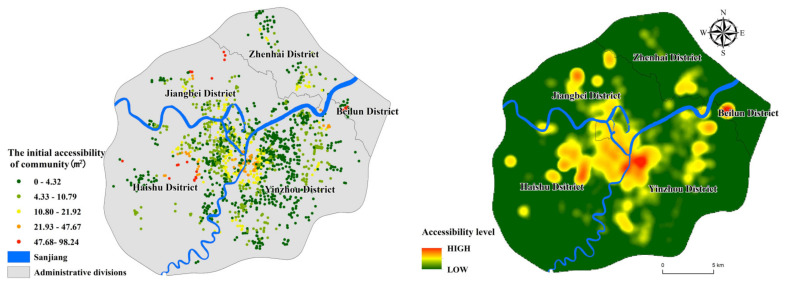
The initial accessibility of residential community.

**Figure 8:**
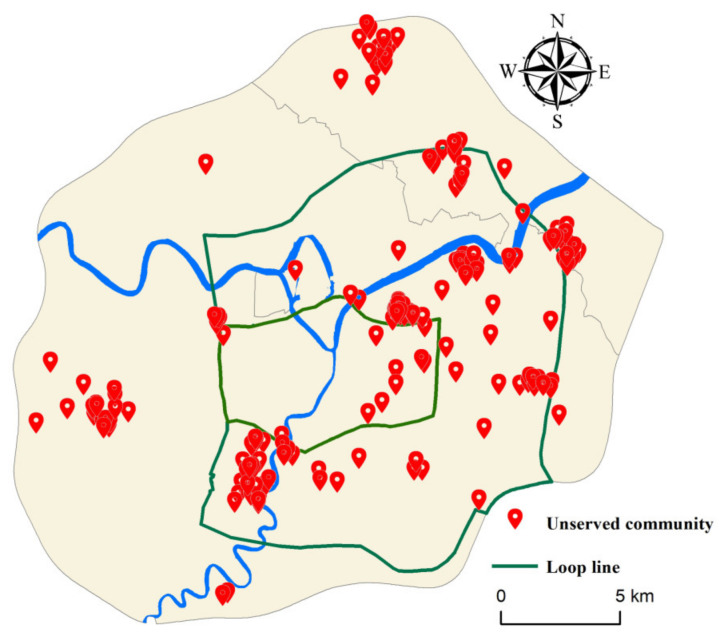
The distribution of unserved communities.

To further study urban parks and residential communities’ spatial and temporal distribution, Ningbo city is divided into three parts: the inner area, middle area, and the outer area (urban-rural junction). We count the year that each residential community was built and compare the time that parks were built. Due to the lack of construction years in some communities, 1514 communities have been counted. Equation (3) is used to calculate PGS accessibility when it has just been constructed (Figure 7). By comparing PGS’s service level at present and the beginning of construction, 181 communities without service were selected, including 11 in the inner area, 113 in the middle area, and 57 in the outer area (Figure 8). Separately calculating the percentage of unserved communities, we found problems in the planning process of PGS in Ningbo.

There are 45 parks in the inner area. The parks were generally built earlier, with the average year of 1997. The number of parks built before 2000 exceeded 20. Early completion of PGS met the needs of residents. The number of communities without service is the smallest; the percentage is only 2.07%. Simultaneously, the number of residential communities in the inner ring has slowed down, and the entire area’s living space is becoming saturated. The residential communities in the inner ring area have been well supplied with infrastructure throughout the city′s development, and residents of this area enjoy some of the best PGS services in Ningbo. The middle area has the most unserved communities with a percentage of 18.61% (Table 2). Over the past 20 years, the number of communities built has maintained rapid growth. There are 43 parks in the area, but only three parks were built before 2000. Although there are sufficient PGS facilities in the middle area currently, many of the residential regions lacked corresponding PGS services at the beginning of construction. This shows that while the middle area is developing rapidly. It does not guarantee the timely matching of the PGS with the community.

The outer area is a typical urban-rural junction, with 57 unserviced communities mainly located in Zhenhai, Beilun, and the west of Haishu. There is a significant PGS vacancy in this area, with nearly one-third of communities not having a corresponding PGS at the time they were built (Table 2). Based on Figure 6 and Figure 7, it can be seen that the ultra-high accessibility residential communities in the outer area did not have corresponding PGS services at the beginning of its construction. Taking Ningbo Zhenhai as an example, the only one comprehensive park in the outer ring area is Ningbo Botanical Garden, which was completed and opened in 2016, while the average construction year of surrounding residential communities was 2010. Although the area currently has good PGS allocation services, there was a severe lack of green space services until 2016. Therefore, the ultra-high accessibility residential communities appearing in the outer area do not enjoy sufficient PGS services in time. And the level of services they enjoy will gradually decline as the population density increases in the future.

Such a result is not found in previous surveys, but it is an element that cannot be ignored. Although the current service level is good, we found that the planning and construction process of green spaces in Ningbo has not been timely in meeting residents’ green space needs in the middle and outer area. There is a shortcoming in evaluating the service level of PGS: it ignores the sequence of residents enjoying green space services. Through the space-time matching evaluation of residential communities and PGS in the central area of Ningbo, we can accurately identify the green space vacant area and the residential community with service vacancy, which provides a more accurate judgment for future targeted solutions.

### 4.3. Comparing the Accessibility of Different Levels of Residential Communitie

In 1998, China implemented the housing commercialization reform. Taking 1998 as the starting year and counting the commercial housing built after that, the socio-economic status of buyers is reflected through house prices. Excluding the houses before 1998 and the houses whose construction year is not available, a total of 1334 commercial communities are counted. According to the average housing price, the residential communities are divided into six levels: first, second, third, fourth, fifth, and unit communities. The higher the group, the lower the income level of the residents of the residential areas. Calculate the average accessibility of each group of residential communities (Table 3).

The results show that: (1) The average accessibility of the unit communities is the highest. At present, the average accessibility of different levels of residential communities in the central urban area of Ningbo is similar, and there is no evident gentrification of PGS. The construction years of the unit communities were all before 1998. They were planned and constructed by the government and distributed in the old city of the Sanjiang area, so they have good green space services. (2) In terms of the average accessibility at the beginning of construction, the unit communities are the highest, followed by the first-level residential communities, and the fifth-level residential communities are the lowest. High-income groups have the smallest difference in PGS accessibility between the two stages, and green facilities can be matched in time. (3) In terms of the number of unserviced communities at the beginning of construction, there is no unserviceable situation in the first and second-level residential communities. Main unserviced communities are concentrated in low—income communities such as the third and fourth levels. High-end residential PGS services have a good match, and they have a priority on enjoying PGS services. There are extensive green space service vacancies in low- and middle-end residential communities. The financial capacity of residents directly affects the level of green space services they receive. Lower-income residents may not have immediate access to PGS when they move into a neighborhood. There are significant social inequalities in the planning process of green spaces in Ningbo.

**Figure 9:**
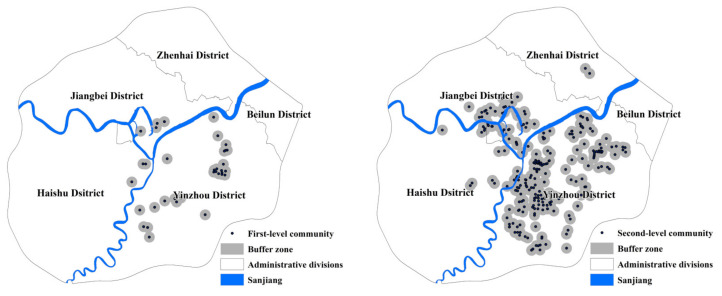

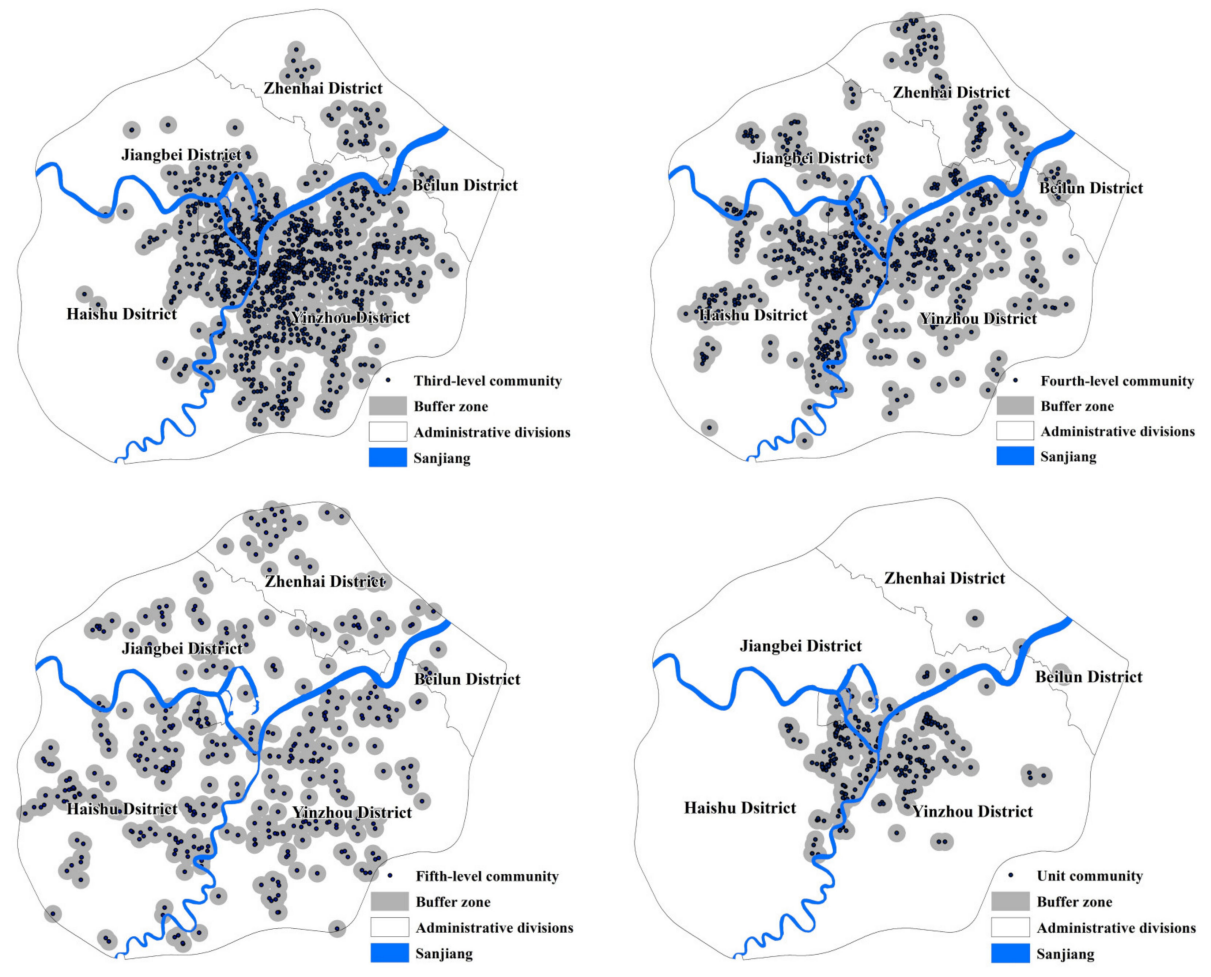
Distribution of different level communities and buffer zone.

We compared the spatial distribution of different residential communities’ levels and the density of the parks (Figure 9). Regarding Ningbo’s requirements for 500 m to see the gardens, 400 m of seeing water, and 300 m to see greenery, set the buffer radius of 500 m. We counted the number of communities within 500 m and calculated the green coverage ratio of various residential areas. It can be seen from Table 4 that the unit community ratio is the highest, followed by the third, fourth, and fifth-level residential communities. The first-level and fifth-level residential communities have the lowest coverage. The number of first-level communities is only 36, of which 21 are villa communities. Compared with traditional high-rise commercial buildings, the villa community has a higher level of green space. The demand for park green space in location selection is significantly reduced, so the coverage ratio is relatively low. However, as the community with the lowest housing price, the five-level community has a scattered distribution, as shown in Figure 7. The communities’ construction is mostly affected by factors such as location and land price, so the coverage ratio is low. We found from Figure 9 that the distribution of secondary, tertiary, and fourth-level communities is concentrated along the Yongjiang, Yaojiang, and Fenghua Rivers. There are also many riverside parks such as Yongjiang Park and Yaojiang Park. The extensive distribution of parks along the river greatly increases the density of parks within 500 m of the community. The distribution of unit communities is concentrated in Sanjiangkou, with the highest rate of seeing garden communities within 500 m.

In general, there is a temporal and spatial misalignment in the planning process of Ningbo Greenland, which leads to PGS inequities. There is a period of obvious green space service vacancies in the middle and low-end communities, and high-income communities have priority in enjoying park services. At the same time, the first-level and fifth-level communities have the lowest percentage of seeing gardens in 500 m.

The first-level communities have natural and superior green space conditions, while the fifth-level communities have much lower percentages than the second, third, and fourth-level communities. It shows that the lowest-income communities have the lowest level of green space coverage, which is unfair. The unit community maintains a relatively high level in terms of accessibility and the ratio of 500 m garden communities. Planners should formulate reasonable greening policies according to the needs of different types of communities to avoid the waste of PGs resources.

## 5. Discussion

The study of PGS in central Ningbo shows that the residential accessibility in the inner ring is higher than that of the middle and outer rings, and that residents in affluent communities benefit more from the green accessibility than those in low—and middle-income communities. This difference is partly attributed to the spatial reconstruction of the green infrastructure in Ningbo. Urban development in the inner ring basically covers all parts of facilities such as medical care, education, and green space. Traffic problems have been alleviated after the opening of urban inner ring viaducts and expressways. Therefore, the urban PGS service level in the inner ring region is the highest. Along with industrial suburbanization, Ningbo takes tertiary industry as the key point of urban development. The development key of the town gradually shifts. The construction of green infrastructure starts, and good green infrastructure in the central area contributes to better accessibility. In the Master Plan of Ningbo city (2006–2020), it is clearly pointed out that the Sanjiang Area need to form a commercial center with dual-core spatial structure and the eastern region should create an ecological leisure axis based on water and green space along the river. In this case, the configuration of urban parks in Sanjiangkou and Eastern New Town is far better than the suburban fringe area.

Our results are similar to those of previous studies, which found that compared with disadvantaged communities, more affluent communities are more likely to acquire green space [51,52]. In the study of urban PGS in Ningbo, it is mainly reflected that the affluent communities have priority in enjoying PGS. However, different from the concept of green gentrification [53], the inequality of green space in Ningbo park is not only reflected in the current distribution of urban green space, but also in the process of PGS configuration in the past 20 years. Affluent community residents have priority in the time sequence of enjoying the park green space service, while the residents in the middle- and low-end communities have a long green space vacancy period. The most affluent communities have special characteristics compared to the above communities. They are located around the Eastern New Town in Yinzhou, which are famous for their villas. The communities are rich in green spaces and have a low demand for PGS. This explains why the richest communities in Ningbo do not have high accessibility and coverage of PGS. This shows that the planners have not implemented appropriate greening strategies according to different type communities.

Managers and planners should be sensitive to the inequalities in urban PGS. The attribute of PGS is not only reflected in the quality and spatial distribution. Besides, the urgency of different social groups′ demands for green space in parks is also an important indicator of green planning. To ensure equal opportunities, PGS services should be provided to the social groups living in the middle- and low-end communities. The spatio-temporal inequality of PGS accessibility in Ningbo can help municipalities and planners determine where further efforts should be made to improve green space equity. The time and space mismatch between housing prices and park green space accessibility help to inspire greening strategies. Planning should provide funds for green space around low-income communities in suburban areas to meet the needs of residents for green infrastructure. We are marking residential areas where green spaces have been vacant in the past in the hope that they will be taken seriously by planners. Meanwhile, in the Special Plan for Ningbo Ecological Green space System, it is proposed that the service radius of park green space 500 m reaches over 90%. As shown in Table 4, there is still a big gap between Ningbo and this target.

This work provides comparative study on the individual accessibility of residential communities so that the method can more accurately evaluate the rationality of urban green space planning. We hope to objectively evaluate the fairness of PGS in this way. Meanwhile, we offer a new object of study for future research. That is to study the micro-perceptions of people in unserved communities. Residents′ perceptions of green space vacancies may not be very sensitive, so our future research will be to investigate residents′ satisfaction with PGS. We would like to know the maximum amount of time residents have allowed PGS vacancies. Setting a maximum threshold for vacancy periods in the future plan can alleviate residents′ frustration with the problem. Although we improve the methods and provide ideas, there are still some limitations. First of all, housing prices are used to reflect the economic conditions of residents without considering relevant factors, including external environmental information such as location, environment, and traffic. In future work, these factors can be combined to reflect changes in socio-economic conditions. Secondly, the green space service level measurement based on the Gaussian-based 2SFCA method takes the spatial distance as the reference, without considering the barrier function of traffic trunk lines, and considering that green accessibility of the Ningbo community may be affected by specific environmental factors, such as the distance to the central business district. It needs to be defined as a control variable in future studies. Thirdly, as for the population of residential areas, there is a certain vacancy rate of houses in the residential areas, so there is a deviation between the population data and the actual situation. We use park green space data, urban block green space and so on are not included in the study. In the future, based on information technology and social software to obtain spatial population data, we should further study the fairness of green space.

## 6. Conclusions

Based on the data of PGS and residential communities, this paper adopts an improved Gaussian-based 2FCA method to calculate the accessibility of PGS in Ningbo. We integrate the year of construction and housing price information to evaluate the equity of PGS in Ningbo. The results show that the accessibility of PGs in Ningbo is good, but there are still regional differences. The middle part of Yinzhou is an obvious low value area, and there are high accessibility communities in the outer ring. By comparing the initial and current accessibility, we found that 181 residential communities with PGS service vacancies. There are significant spatial differences with initial PGS accessibility in the middle and outer Rings. The current high accessibility communities in outer ring lack PGS service at the beginning of their construction. Unserved communities are concentrated in low-end and mid-range neighborhoods. High-end communities have absolute priority access to the park green space service sequence. The highest end and lowest end communities have the lowest percentage of garden visits at 500 m. The most expensive communities have a very privileged natural environment inside and they don′t need PGS services. While the lowest end of communities have the lowest level of green area coverage, which is unfair.

In the past, Chinese residents generally paid more attention to educational resources, commercial facilities, and transportation facilities. With the improvement of living standards, they care more about their health and PGS facilities have also attracted more and more attention. The evaluation of PGS accessibility based on spatial distribution cannot accurately identify communities with green space vacancies, which will impact future in-depth research. Government planning could lead to 5–10 years of residents not getting PGS services. This is unacceptable for a family. Therefore, it is very necessary to identify these groups, because they may dominate the future urban green space planning. That is why we do this research. Our research has identified these groups and demonstrated that social inequality is the main cause. In the future, planners may not be able to avoid this phenomenon. Therefore, future research will focus on the perception of green space among residents of unserved communities. Based on the discussion and conclusion of our study, we put forward the following relevant policy suggestions to the future planning: (a) Adjust the allocation of resources according to the needs of the population, focus on vulnerable areas, and add new PGS. We suggest to create a new comprehensive park in middle area of Yinzhou to meet the recreational needs of a large population. (b) The amount of green space within different communities varies, thus planners should implement appropriate greening strategies for different type communities. (c) Gaining awareness to the green space vacancy in the process of planning, the people without park green space service can provide meaningful ideas for the future planning.

## Figures and Tables

**Table 1:** Park level and service supply radius.

Type	Green Area/hm^2^	Service Radius/m
Comprehensive park	≥0.5	5000
	20~50	3000
	10~20	2000
Community park	3~10	1200
	1~3	800
Recreational garden	≥0.5	500
	0.1~0.5	300

According to China’s “Standard of Urban Green Space Classification”.

**Table 2:** Number and percentage of unserved communities.

	Inner Area	Middle Area	Outer Area
Unserved communities	11	113	57
Percentage	2.07%	18.61%	29.53%

**Table 3:** Accessibility for residents of various houses.

Year of Construction	>1998					≤1998
Residential Community Level	First	Second	Third	Fourth	Fifth	Unit Communities
Price (￥/m^2^)	48587–114240	32811–48586	25053–32810	18126–25052	7244–18125	
Resident attributes	High	Mid to high	Medium	Mid to low	Low	
Current accessibility	10.292574	9.008513	11.430455	13.258195	10.863257	15.878173
Initial accessibility	9.528793	7.879245	8.07011	8.496531	7.479777	10.738079
Unserved communities	0	2	44	85	23	27

**Table 4:** The proportion of 500 m see gardens in residential communities.

Community Level	First	Second	Third	Fourth	Fifth	Unit Community
Number	36	251	1088	728	382	254
Parks in the buffer	5	48	87	74	54	43
Coverage ratio (Cr)	0.194	0.310	0.406	0.351	0.196	0.543
